# Notes for Contributors

**DOI:** 10.1155/S1463924678000024

**Published:** 1978


### Commentary

### Automation comes of age

The publishing of this Journal represents the beginning of the end; the end of a struggle to legitimise automation in chemistry and the beginning of what I trust will be a valuable means of communication for those of us, as chemists, who become increasingly involved with automatic analysis.

Automation can be defined in many ways but should not be used simply to imply mechanisation of a single facet of an analytical measurement or computing. Automation is a systems problem which must be applied at all levels of the analytical process from measurement through to data acquisition and analysis.

Whilst automation has been applied to process control situations for a long time it is undeniable that the acceleration in acceptance of automation stems from the significant introduction, in the clinical area, of the concept of continuous flow analysis. The rapid developments of computers and electronics coupled with the more recent emphasis on microelectronics has generated much interest from commercial manufacturers. Automation has been applied to almost all areas of chemical analysis. Its influence therefore crosses disciplinary boundaries, but perhaps more significantly the influence of automation transcends the science involved, impinges on the role of management and on the structure of organisations and also has economic connotations. Research and development has been carried out by many groups and the results are published, if at all, in a variety of journals. Often, because the work reflects a study of economic or organisational problems they are not published. This poses a problem of communication and no journal has catered for this need. The *Journal of Automatic Chemistry* has been conceived to repair this gap in the literature. The response to the initial announcement has further consolidated my view that it is indeed timely to publish such a journal. Some 21 years after the introduction of Skeggs' AutoAnalyzer, automation has at last come of age and is a subject in its own right.

The joint involvement of clinical chemistry, industrial chemistry and analytical chemistry generally is not an affair to be considered lightly, but hopefully the problems of automation are so similar as to unite the various groups in common effort.

The contents of this first issue have been drawn from across various disciplines and in addition have come from academic, clinical, industrial, commercial and government institutions. The articles include detailed technical papers, results of evaluations and short technical notes. As time progresses the balance between these articles and other editorial features no doubt will change to meet the needs and requests of *you* the readers. The overriding aim is to provide an effective and efficient means of communication in the area of automatic chemical analysis.

Within the UK, a major interest is centred on microprocessors; this I feel distorts the balance of effort in the field of automation. Microprocessors are just tools that the systems designer can use effectively and efficiently if they are required. Microprocessors are certainly no substitute for a proper specification of the analytical problem and the chemistry involved in automation.

I am particularly encouraged at the response to the concept of the *Journal of Automatic Chemistry* and by the stature of those people who have been enthusiastic to be involved in an editorial capacity. The Editorial Board of Rolf Arndt, Howard Malmstadt and Fred Mitchell represents a wealth of experience and knowledge on automatic analysis. The Board members will direct the editorial policy in close co-operation with myself and the publisher. In addition, the Corresponding Editors have been drawn from a variety of backgrounds and their origins fully represent one basic aim of the journal, to be *international*.

Your views and comments on the content and format of this journal and on automatic chemistry, generally, would be welcomed.

*Peter B. Stockwell*

### Notes for Contributors

### Presentation of manuscripts

Manuscripts should be typed (double-spaced) on one side of the paper only and with generous margins. The title should be brief and informative avoiding the word "new" and its synonyms. The full list of authors with their affiliations and full address(es) should appear on the title page. On a separate sheet an abstract of no more than 150 words is required. This should succinctly describe the scope of the contribution and highlight significant findings or innovations. It should be written in a style which can easily be translated into French and German.

*The Concise Oxford Dictionary* and Fowler's *Modern English Usage* (both published by Oxford University Press) should be used as the standard for spelling and grammar. Abbreviations should be limited to those generally recognised, or where a frequently occuring term is abbreviated it should, in the first instance, be explained thus "flow injection analysis (FIA) ..." and the abbreviation used thereafter. Abbreviations for standard measures and units should follow SI recommendations. There are various publications giving guidance on the use of SI units.

References should be indicated in the text by numerals following the author's name, i.e. Skeggs [6]. On a separate sheet of paper, list all references in numerical order thus:

[6] Skeggs, L. T., *American Journal of Clinical Pathology*, 1959, **28**, 311.

Note that journal titles are given in full. Where there is more than one author, the form Foreman et al. should be used in the text but all authors should be named in the list of references.

When reference is made to a chapter in a book the reference should take the following form:

[7] Malmstadt, H. V. in "Topics in Automatic Chemistry" Ed. Stockwell P. B. and Foreman J. K. 1978 Horwood, Chichester, pp. 68-70.

Only work which has been published or has been accepted for publication should be cited. Avoid the citation of documents which are subject to restricted circulation, patent literature, unpublished work and personal communications. The latter can be mentioned in the text in parenthesis.

To illustrate a paper line diagrams are preferred to photographs. Photographs should only be used when they significantly add to the discussion. Diagrams, charts and graphs should be carefully drawn in black ink on stout card or heavy quality tracing paper. Most illustrations are reduced for publication; to allow for this originals should be between 16 and 36 cm wide (the depth must not exceed 50 cm). The lettering of diagrams should be sufficiently clear to withstand reduction. Except in the case of proper names, all lettering should be in lower case print. If photographs are used they must be supplied in the form of clear, unmounted, glossy, black and white prints. "Instant" photographs are not normally acceptable. All illustrations must be identified on the reverse showing the figure number and the author's name.

Each illustration should have a fully explanitory caption. Captions should be typed together on a separate sheet of paper; they must *not* be an inseparable part of the illustration.

